# The Prevalence of Atopy in Biologically Treated Spondyloarthropathies: A Retrospective Study of 200 Patients

**DOI:** 10.3390/jcm11010055

**Published:** 2021-12-23

**Authors:** Georgiana Strugariu, Cristina Pomîrleanu, Codruța Bran, Andrei Costea, Andrei Vicovan, Diana Tatarciuc, Irina Eșanu, Eugen Ancuța, Rodica Chirieac, Codrina Ancuța

**Affiliations:** 1 Rheumatology Department, University of Medicine and Pharmacy “Grigore T Popa”, 700115 Iași, Romania; georgiana_strugariu@yahoo.com (G.S.); codrina_ancuta@yahoo.com (C.A.); 2 2nd Rheumatology Department, Clinical Rehabilitation Hospital, 700664 Iași, Romania; 3 Faculty of Medicine and Biological Sciences, Ștefan cel Mare University of Suceava, 720229 Suceava, Romania; codruta.bran@usm.ro (C.B.); drandreicostea@gmail.com (A.C.); 4 Department of Morpho-Functional Sciences II—Pharmacology and Clinical Pharmacology, University of Medicine and Pharmacy “Grigore T Popa”, 700115 Iași, Romania; andrei-gheorghe.vicovan@umfiasi.ro; 5 Department of Internal Medicine and Geriatrics, University of Medicine and Pharmacy “Grigore T Popa”, 700115 Iași, Romania; diana.tatarciuc@umfiasi.ro (D.T.); irina.esanu@umfiasi.ro (I.E.); 6 Research Department, “Elena Doamna” Clinical Hospital, 700398 Iași, Romania; 7 SANOCARE Medical and Research Center, 700259 Iași, Romania; chiriac01ro@yahoo.com

**Keywords:** spondyloarthropathies, atopic disease, allergic rhinitis, allergic asthma, atopic dermatitis, biologic therapy

## Abstract

(1) Background: Recent data shed light on the association between atopic disorders (ADs) (atopic dermatitis, allergic asthma, allergic rhinitis) and spondyloarthropathies (SpAs), underpinning the critical role of T helper (Th)1-Th17/Th2-T regulatory cells disbalance. We evaluated the prevalence of AD in axial SpAs (axSpAs) and psoriatic arthritis (PsA) and explored the potential association between atopic status, disease-related parameters, and biological therapy. (2) Methods: A monocentric, retrospective study was conducted that enrolled 200 patients taking biologics. Demographics, disease, and drug-related variables, along with a screening questionnaire focused on Ads, were systematically collected. (3) Results: Overall, 51 patients (25.5%) had atopy—namely, 24.4% of axSpA and 28% of PsA, with a higher frequency of rhinitis (43%) vs. atopic dermatitis (37.2%) or asthma (21.5%). We failed to demonstrate any statistically significant difference in demographics, SpA-related parameters excepting concomitant inflammatory bowel disease, and biologic drug exposure in patients with and without atopy (*p* > 0.05). However, significantly more non-atopic patients need only one TNF inhibitor (54%) vs. atopic patients (28%) (*p* < 0.05) to control active SpA. (4) Conclusions: We successfully demonstrated that AD is associated with one out of four SpA. Irrespective of the SpA subtype, atopic patients require more frequent switching among biologics, as significantly more non-atopic patients remain on their first anti-TNF.

## 1. Introduction

Spondyloarthropathies (SpAs) comprise a multifactorial and heterogeneous group of chronic immuno-inflammatory rheumatic disorders primarily affecting the axial skeleton, but also presenting with peripheral (joints and enthesis) symptoms, as well as systemic damage (eye, heart, gut) [1,2]. Owing to the high clinical variability, SpAs are currently stratified into axial SpA (axSpA) spectrum, including non-radiographic axSpAs (nr-axSpAs) and radiographic axSpAs or ankylosing spondylitis (AS), and peripheral SpAs (pSpAs), consisting of psoriatic arthritis (PsA), SpAs associated with inflammatory bowel disease, reactive arthritis, and undifferentiated conditions [2,3]. Overall, the complex and dynamic pathobiology of SpAs focuses on a T helper (Th)1/Th 2 imbalance with Th2 polarization and on the distinct intervention of Th17 cells in both articular and entheses inflammation, as well as osteoproliferative lesions labeled in AS and PsA [2,4,5,6]. Recognized as one important proinflammatory cytokine involved in early local and systemic inflammation of SpA, tumor necrosis factor (TNF)α remains a key player, overexpressed by Th1/macrophage axis, which is activated by interleukin (IL)-12 and IL-23 [1,4,5,6]. On the other hand, Th2 cells are also activated in SpAs, secreting anti-inflammatory IL-4, IL-10, and IL-13 but holding the ability to modulate, or even inhibit, Th1, and to activate macrophages and lymphocytes B [1,2,4]. Moreover, in an attempt to restore balance, Th2 has the ability to activate the Th17 axis, both Th2 and Th17 being found in increased amounts in the serum of patients with SpAs [2,4,5,6].

In recent decades, few papers have raised attention on the association of atopic disorders (ADs), particularly atopic dermatitis, allergic asthma, and allergic rhinitis, with immuno-inflammatory diseases including SpAs, through the common pathogenetic pathway of the IL-17/IL-23 axis with Th2 disbalance and high immunoglobulin E serum concentrations [6,7,8,9]. About 30 years ago, Rudwaleit et al. (1992) indicated a higher prevalence of atopy in AS patients, compared with seropositive rheumatoid arthritis (RA) [9]; since then, several large studies and case reports suggested an immunological link between SpAs and atopic conditions, which is worth to be analyzed in order to better understand the complexity of the immune system and find better therapeutic molecules for these patients [10]. The explanation on why patients with AS but not those with RA would have a higher rate to develop atopy resides in the Th1/Th2 paradigm which was recently updated and extended to two new players—namely, T regulatory cells (Tregs) and Th17 cells, was described [7,9,10,11]. TCD4 + Treg cells are able to prevent immune responses against self-antigens and allergens by inhibiting both Th1 and Th2 cells, while Th17 and their proinflammatory IL-17 family are activated by abnormal Tregs [11]. The naturally occurring Treg (nTreg) subset contributes to prevention in autoimmune conditions, whereas the adaptive or inducible subset (iTreg), especially IL-10-producing type 1 Treg, has a well-documented role in both allergy and autoimmunity [11].

Atopic disorders share the same immune background governed by Th2 and Th17 cells and their cytokines, particularly IL-3, IL-4, IL-5, and IL-13, which actively participate in the humoral and atopic responses [11,12]. In addition, proinflammatory Th17 cells and inducible Th2 cells able to synthesize excessive TNFα amounts are upregulated during late stages of atopic conditions resulting in symptomatic allergies [11]. Conversely, patients with asymptomatic atopy had increased number and activation of rTh2, another Th2 subpopulation, able to produce high quantities of anti-inflammatory IL-10 and to promote Th17 cells downregulation [11]. A closer look at the pathways of different atopic conditions suggests that specific mechanisms endorse their immunopathogenesis. Thus, allergic asthma is typically related to Th17 cells expressing several effector cytokines such as IL-17A and 17F, IL-22, IL-26, and granulocyte-macrophage colony-stimulating factor (GM-CSF) [13]; conversely, atopic dermatitis is clearly a Th2-type-driven inflammation, while allergic rhinitis is classically defined by the imbalance between Th1/Th2 response, with a predominance of Th2 and Th17 cells [14,15,16].

Both SpAs and ADs are, therefore, caused by a defect in the immune system at some point during the lifetime, which promptly shifts the immune cell CD4+ pattern by activation of specific T helper subpopulations. When activation of the Th2 pattern is “on”, an atopic profile is shaped, while the activation of the Th1 population and overexpression of proinflammatory cytokines (INFγ, IL-2) endorses a non-atopic profile potentially advocating for autoimmunity [7,12,16]. This tight link between Th2, Th1, and Th17 may explain why atopic diseases, SpAs, and other immuno-inflammatory disorders can be connected [2,12,16].

Biologic disease-modifying antirheumatic drugs (bDMARDs) addressing potent inflammatory cytokines such as TNFα and IL-17 have radically changed the outcomes of SpAs, remission, or low disease activity being achievable in routine practice in most patients [17,18]. Moreover, there are clinical studies that analyze the efficacy of biological treatment in atopic disorders [19].

While several reports emphasized that patients with AS and PsA carry a higher risk for concomitant atopic diseases, particularly allergic rhinitis and asthma [8,9], data on how drugs targeting inflammatory cytokines may influence the atopy in patients with SpA are still controversial. Indeed, in their 10-year follow-up cohort study conducted in an Asian population, Chang et al., (2016) have demonstrated an increased risk of allergic diseases in AS; additionally, the authors raised the question about how DMARDs may influence the expression of atopic disorders in such patients, since biologic agents, particularly TNF inhibitors (TNFis), might affect the Th1/Th2 balance [8,17]. Impaired Th1 cytokine production in SpAs is restored by anti-TNFs, as demonstrated in 2001 by Baeten et al., who evaluated the effect of TNFi on Th1 and Th2 cells in AS patients [17].

The current study aimed to evaluate the prevalence of asthma, atopic dermatitis, and allergic rhinitis in patients with axSpA and PsA and to explore any potential association between atopic status, SpA-related parameters, and biological therapy.

## 2. Materials and Methods

We conducted a retrospective analysis in a cohort of 200 consecutive patients with different spondyloarthropathies (non-radiographic axial SpAs, ankylosing spondylitis, psoriatic arthritis) who are currently taking biologics (TNF and IL-17 inhibitors) to control the active disease. Their participation in the current study was based on the agreement to respond to a screening questionnaire focused on atopy, applied once between January 2020 and July 2021; moreover, eligible patients had to receive their biological treatment continuously, with no gaps in routine during the 6-month follow-up in our clinic.

Participants were selected from a total of 389 patients with SpAs treated with biological therapy and who followed up between 2010 and 2021 in one academic rheumatology department in northeastern Romania. The remaining 189 patients were not eligible since they were no longer routinely monitored in our center or no longer under biological treatment due to diverse reasons (dropouts since the previous years, dropouts due to the COVID-19 pandemic, or referred to other clinics in locations more closely to their homes).

The diagnosis, classification, and management of the full spectrum of SpA were in line with expert recommendations and consensus guidelines European Alliance of Associations for Rheumatology (EULAR) and Romanian National Protocol for the treatment of axSpAs and PsA [20,21,22,23].

A brief synthesis of our local guidelines for biological therapy in axSpAs focus on several concomitant entry criteria as follows: (i) positive diagnosis of axSpA using either modified New York criteria (for AS) or ASAS criteria; (ii) active and severe disease as assessed by a Bath Ankylosing Spondylitis Disease Activity Index (BASDAI) score ≥ 6 at two additional visits, at least 4 weeks apart, and Ankylosing Spondylitis Disease Activity Score (ASDAS) ≥ 2.5, together with parameters for systemic inflammation: erythrocyte sedimentation rate (ESR) > 28 mm/h and/or C reactive protein (CRP) > 3 times normal upper limit; (iii) sub-optimally controlled disease by traditional treatment meaning at least two non-steroidal anti-inflammatory drugs (NSAIDs) for at least six weeks continuously each, at maximal recommended or tolerated doses OR sulfasalazine 2–3 g/day at least 12 weeks for peripheral involvement. Similarly, patients with PsA were potential candidates for bDMARDs if they meet four concomitant criteria: (i) a definite diagnosis of PsA according to the Classification Criteria for Psoriatic Arthritis (CASPAR); (ii) severe PsA with high disease activity as definite by a Disease Activity in Psoriatic Arthritis (DAPSA) score > 28 despite conventional immunosuppressive drugs; (iii) failure to at least two conventional synthetic drugs meaning persistent active disease after 12 weeks with the maximum recommended doses, except for patients with predominantly axial PsA and those with active enthesitis and/or dactylitis, in which the use of NSAIDs in the maximum doses in the last 12 weeks is sufficient.

Exclusion criteria or contraindications to biologics comprised latent or active chronic infections, severe chronic cardiac failure, history of or active neoplastic disorder.

Drugs reimbursed as per local regulations include all five TNF inhibitors (TNFis)—original and biosimilars of infliximab (IFX), original and biosimilars of adalimumab (ADA), original and biosimilars of etanercept (ETA), golimumab (GLM), and certolizumab pegol (CZP)—as well as the more recently approved IL-17 inhibitor (IL-17i), secukinumab (SEK).

### 2.1. SpA-Related Data

For this study, we collected demographic data, disease-related parameters (a subset of SpA; type of manifestations—axial, peripheral, extra-articular; disease duration; disease activity scores), and drug-related parameters (type of bDMARDs; years of persistence on a specific drug; the number of switched bDMARDs).

### 2.2. Atopic Disorder Data

We applied a detailed screening questionnaire (the so-called 5Q) comprising five standardized questions specifically designed for the identification of atopy, addressed either during routine on-site visits or by phone in every enrolled case (Table 1). Each question had a dichotomous answer—“yes” or “no” and patients were invited to select the answer most correctly describing their situation. In the atopic group, we included all SpA cases who positively answered “yes” to Q1 and Q2 but not those who selected “yes” for Q5 and “no” for Q1 and Q2. Moreover, Q3 and Q4 were introduced to optimize our knowledge about the potential influence of biological treatment on signs and symptoms of atopy.

Furthermore, data on allergic asthma, allergic rhinitis, and atopic dermatitis were also collected, including time of diagnosis (during childhood (early atopy) or adulthood (late atopy)), before or after the diagnosis of SpA, key manifestations, worsening or improvement (even complete resolution) under biologics.

All patients enrolled in the study came from the northeastern counties of Romania, having the same climate, food, or living traditions known as potential confounders in atopy.

Before the initiation of biological therapy, patients were required to sign informed consent as per local procedures, while they also gave written consent for the current study. The study protocol received the approval of local ethics committees (No. 42/02.2021).

### 2.3. Statistical Analysis

Statistical analyses were performed using the OPENSTAT (William G. Miller, Ames, IA, USA) software; all variables had a non-parametric distribution, as demonstrated by Shapiro-Wilk and Lilliefors tests; Mann-Whitney and chi-squared were used in the subgroup analysis (SpA with and without atopy), and a statistically significant *p*-value was defined as <0.05.

## 3. Results

### 3.1. Demographic Data, Disease, and Treatment-Related Parameters

Table 2 shows the distribution of main demographic characteristics, disease, and treatment-related parameters in our cohort.

#### 3.1.1. Demographics

Out of 389 patients, 200 patients with a diagnosis of SpA who are prescribed biologics in our database were eligible and enrolled in the current study, comprising 143 (71.5%) axSpA (AS, nr-axSpA) and 57 (28.5%) PsA patients. The mean age was 48.7 years, and the mean duration of the disease was 15.69 years (1–37 years), with a mean delay of diagnosis (the latent period is between first symptoms of disease and positive diagnosis of SpA) of 3.18 years (0–33 years). As expected, the majority of axSpA patients were male (78.3%), with an urban lifestyle (58%) and younger than those enrolled in the PsA cohort (83.8% are under 60 years old) (*p* < 0.05). On the other hand, in the PsA cohort, patients were almost equally distributed among genders (46% female), and 91% were non-smokers (*p* < 0.05).

#### 3.1.2. Disease-Related Parameters

Detailed subgroup analysis was further performed. The axSpA patients are distributed as follows: 131 (65%) AS cases, including those with AS associated with inflammatory bowel disease (6 cases, 3%), and 12 (6%) cases with non-radiographic axSpA; more than half (84 cases, 58.7%) presented also peripheral manifestations. Among PsA patients, 41 (72%) had symmetrical polyarticular involvement, 14 (24.5%) axial involvement, and 2 (3.5%) arthritis mutilans. We reported a statistically significant difference (*p* < 0.00001) when comparing axial and peripheral involvement between subgroups.

#### 3.1.3. Biologic Drugs

Incontestably, the majority of cases in our cohort were on TNF inhibitors (mainly adalimumab, etanercept, infliximab, but also the newer agents, e.g., golimumab and certolizumab) since this class of biologics was, for many years, the only reimbursed by our health insurance. Thus, 157 (78.5%) patients were prescribed a TNFi, 40.1% adalimumab, 35.03% etanercept, 9.5% certolizumab, 8.2% infliximab, and only 5.7% golimumab; among them, 89 (56.7%) were still on their first biological agent, while 68 (43.3%) changed between two and five TNF inhibitors.

Only 43 patients (21.5%) were taking IL17 inhibitors, 21 cases (48.8%) 150 mg monthly secukinumab (18 AS, 3 PsA) and 22 (51.2%) patients the full dose of 300 mg (18 PsA, 4 AS). In addition, we reported a mean duration since the first administration of biological drugs of 6.6 years (1–18 years) for TNFi and only 0.44 years (1–3 years) for IL17i, explained by the relatively recent approval of anti-IL-17 agents for the treatment of active SpA and local reimbursement starting from 2017.

### 3.2. Prevalence of Atopy

**Figure 1:**
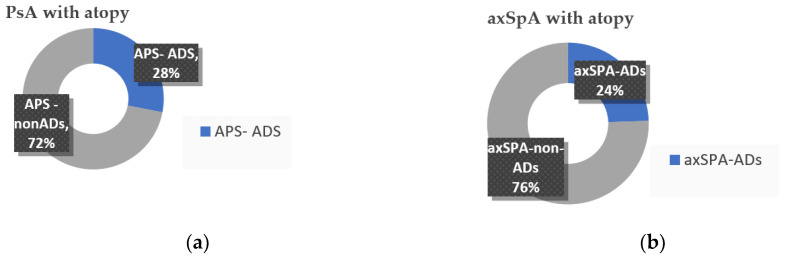
SpA patients with and without atopy: (**a**) PsA; (**b**) axSpA; ADs, atopic disorders.

We stratified patients in atopic and non-atopic SpA based on their answers on the 5Q screening questionnaire; the atopic group comprised all patients who answered “yes“ in Q1 and Q2, meaning that they had or still have atopy (current atopy) diagnosed and followed up by a specialist. Overall, atopic disorders were reported in up to 51 (25.5%) patients of our cohort; among them, 37 cases (72.5%) presented with a form of atopy in their medical history before SpA was diagnosed, while 14 patients (27.4%) were diagnosed with atopy during the evolution of SpA. Conversely, patients who positively answered to Q5 (meaning that they have an allergy to drugs or other external chemical substances and no other atopic disorders) were excluded from the atopic group. Overall, 24.4% of axSpA and 28% of PsA patients have atopy; there is no statistically significant difference between the proportion of patients with atopy in subgroup analysis, as calculated by using the chi-squared test (*p* = 0.598) (Figure 1).

**Figure 2:**
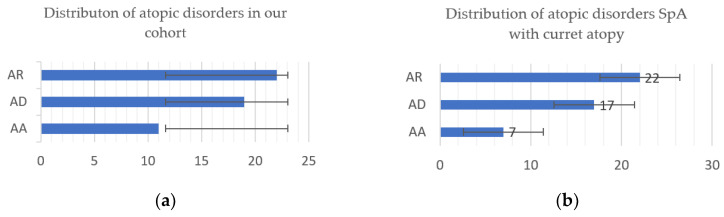
Distribution of atopic disorders among SpA with atopy (**a**) and SpA with current atopy (**b**) (AR, atopic rhinitis; AD, atopic dermatitis; AA, allergic asthma).

We further assessed the percentage of patients with different subtypes of atopy, taking into account all 51 patients with SpA and atopic symptoms sometimes during their life. Some of them have overlapping two atopic disorders, such as allergic rhinitis and atopic dermatitis or rhinitis and asthma. Hence, when analyzing different atopic conditions separately, we reported a higher frequency of rhinitis (22, 43%) versus atopic eczema (19, 37.2%) or asthma (11, 21.5%) (Figure 2a). A closer look showed that 46 patients who positively answered questions Q2, Q3, and Q4 have current atopy: 7 patients under concomitant treatment for asthma, and 17 with intermittent symptoms of atopic dermatitis controlled with topic treatment and/or antihistaminic drugs, while 22 with allergic rhinitis required constant treatment. Three patients declared symptoms of at least two atopic disorders (Figure 2b).

### 3.3. Atopy and SpA (Disease-Related Parameters and Biologic Drugs)

In the second step, we were interested in evaluating the atopy in relation to different parameters including gender, age, lifestyle, smoking status, key clinical manifestations (skeletal and extra-skeletal), disease duration, and persistence on TNFi and IL-17i (Table 3).

We failed to identify any significant difference in demographic variables between atopic and non-atopic cohorts (*p* > 0.05, chi-squared test). Furthermore, there were no statistically significant differences (*p* > 0.05) in axial and peripheral involvement between atopic and non-atopic patients: 73% axial and 62% peripheral manifestations in the atopic cohort, as compared with 96% axial and 56% peripheral involvement in non-atopic SpA, respectively.

However, we reported significant differences only in concomitant inflammatory bowel disease, 7 out of 12 patients with intestinal involvement being among atopic SpA (*p* = 0.007); intestinal involvement could be an independent factor for atopy, and further studies are required to assess the link between the intestinal microbiome and atopy mechanisms in patients with SpAs. Conversely, ocular involvement was not evaluated since axSpAs a priori develop more acute anterior uveitis than PsA and we enrolled predominantly axSpA patients in our cohort.

Additionally, we could not demonstrate any significant difference between mean disease history, delay from onset to diagnosis, and the number of years under biological treatment in atopic SpA, compared with non-atopic SpA (*p* > 0.05). One potential explanation relies on eligibility criteria for biologics according to local recommendations comprising long-term history, SpAs incompletely controlled by classical medication, and multiple negative prognostic factors.

Furthermore, SpAs with and without atopy have the same global persistence on biologics; mean exposure to TNF biologics was 6.9 years (median of 8 years, range between 1 and 16 years) for atopic SpAs and 6.5 in non-atopic SpAs (*p* > 0.05), and the same mean exposure of 0.4 years (1–3 years) for atopic and non-atopic SpAs (*p* > 0.05) for IL-17 inhibitors.

**Figure 3:**
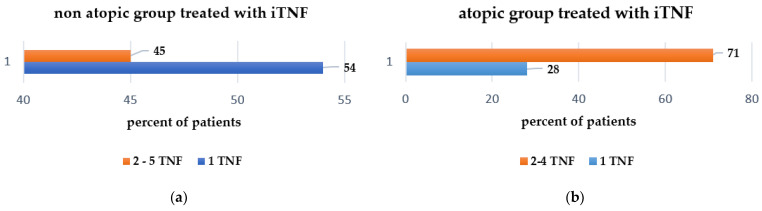
Number of TNF inhibitors used to treat SpAs patients: (**a**) non-atopic cohort; (**b**) atopic cohort.

**Figure 4:**
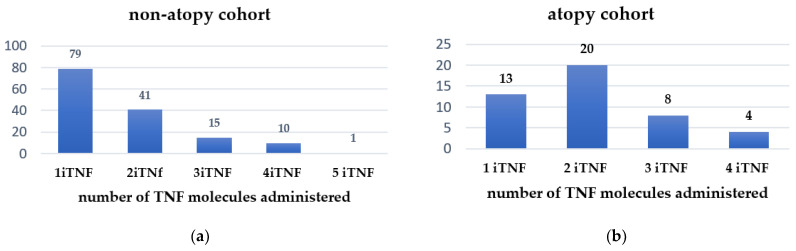
Number of TNF inhibitors used to treat SpA patients: (**a**) non-atopic cohort; (**b**) atopic cohort.

In total, 67 (46%) non-atopic SpA patients were classified as non-responders in more than two anti-TNF agents, and 79 patients (54%) in this group required only one TNF inhibitor to control disease activity (Figure 3a). We were also interested in evaluating the number of switching required to control SpA activity—namely, 20 patients (39.2%) in the atopic cohort switched at least one TNF inhibitor and 12 patients (23.5%) needed more than three anti-TNF molecules, while 25.5% (13 patients) only one anti-TNF in order to attain sustained remission or, at least, low disease activity (Figure 3b). Details on the number of TNF inhibitors administered for each cohort (atopic and non-atopic) are provided in Figure 4 and Table 4 clearly shows that significantly more patients in the non-atopic cohort (54%) are controlled by their first TNF inhibitor, compared with those in the atopic cohort (28%).

SpA patients with atopy required 1.92 TNF inhibitors (median od 2, range 1–4 molecules) versus 1.7 for SpA patients without atopy (*p* > 0.05). Although statistically non-significant, there is a numeric difference between patients taking 300 mg monthly of secukinumab (13 atopic patients versus 28 non-atopic patients). Further studies are necessary with comparable cohorts of patients (number, disease subtype, exposure period) to evaluate the influence of IL-17 inhibitors (drug, dose) on atopic symptoms.

Patients with atopy also needed a higher dose of secukinumab in 25.5%, as shown in Table 3.

## 4. Discussion

The current study was specifically designed to assess the prevalence of atopic disorders among patients diagnosed with SpAs (either axSpA or PsA) and ongoing biological therapy and to identify potential correlations between atopy and demographic data, disease, and bDMARD-related parameters in a cohort of 200 patients.

Despite emerging data on cytokine patterns in AS and various atopic conditions such as asthma, atopic dermatitis and allergic rhinitis, the association between atopy and immune-mediated rheumatic conditions is still controversial. Indeed, different studies have underpinned the mutual inhibition of Th1 and Th2 axes, suggesting that Th1 and Th2 polarized diseases exclude each other, e.g., atopy (Th2 polarization) provides protection for the development of rheumatoid arthritis (Th1 polarization) and vice versa but associates with ankylosing spondylitis (Th2 polarization) [7,8,9,12,24].

Rudwaleit et al., (2002) have analyzed the risk of atopy in Caucasian patients with rheumatoid arthritis or ankylosing spondylitis; overall, atopic disorders were more prevalent in AS (24.6%), compared with seropositive RA (13.1%), but non-significantly increased, compared with controls (20.7%). We have also shown comparable data—one in four patients with SpA and up to one-third of those with PsA have a concomitant atopic disorder in our monocentric retrospective cohort; these results are also similar to those reported in the literature.

When analyzing different atopic conditions separately, the prevalence of asthma, allergic rhinitis, and atopic dermatitis were highest in AS, intermediate in controls, and lowest in the RA population [9]. Moreover, patients with AS have a 1.31 times greater risk of developing asthma, 1.46 times higher risk for allergic rhinitis, and 1.22 times for atopic dermatitis, compared with the general population, according to the results published by Chang et al. (2015), in a 10-year follow-up, population-based study in Taiwan [8]. Data are confirmed by Shen et al. (2015), who documented the increased risk of allergic asthma in AS under various pathogenic medications, regardless of age and sex, also in an Asian cohort [25]. A recent study suggests that atopy is not commonly associated with psoriasis vulgaris, supporting the concept that Th2-mediated atopy protects against certain Th-1-mediated autoimmune pathologies such as psoriasis, in which we discussed the intervention of the proinflammatory cytokine axes TNF, IFN type 1, and IL-17 [14,26].

We demonstrated a higher prevalence of allergic rhinitis (54%), compared with atopic dermatitis (35%) and allergic asthma (20%) in our cohort, similar to data reported in other studies that showed a higher incidence of rhinitis in SpA patients [10].

A closer examination of the potential correlation between atopy and gender, age, disease duration, and severity revealed that all patients in our atopic cohort were mainly male (70%), older than non-atopic patients, aged more than 35 years old (40–60 years), and more than half featuring an urban lifestyle. Conversely, Chang et al. (2018) demonstrated a significantly higher incidence of AD in patients with AS younger than 20 years old, and a trend of a higher incidence rate of allergic diseases in different age groups; however, the authors point to the small case numbers in each age group, suggesting that it may not be powered to detect the difference. Moreover, they recommended monitoring related signs and symptoms of atopic dermatitis in younger patients with AS [8].

Overall, it seems that the prevalence of atopic disorders in our cohort is comparable irrespective of the activity and management, suggesting that the biological therapy does not change the frequency of atopy. In other words, concomitant atopic conditions remained unchanged even after the initiation of bDMARDs in patients with a well-defined atopic status. Moreover, we could not identify any correlations between persistent atopic disease and negative prognostic factors for SpA, theoretically related to specific criteria for the access to biologics in our country that result in a homogeneous population. Only one factor could potentially influence the clinical significance of atopy in our patients, and this is related to the concomitant inflammatory bowel disease—namely, 14.5% of cases in the atopic subgroup were associated with intestinal inflammation, three times higher than the non-atopic patients.

To the best of our knowledge, this is the first analysis of atopic disorders among severe active SpA taking biologics in a Romanian population. Despite discrepancies between subgroups (number of patients, rate of TNFi users compared with IL-17i users, exposure to TNFi versus IL17i), one major advantage of the study is the relatively homogeneous population in terms of SpA activity, as biologics are prescribed according to well-established local recommendations.

One potential limitation of the current study is that the majority of patients in our cohort had a long history of their SpAs (a mean of 15 years), with a mean exposure to anti-TNF agents for more than six years; since the persistence on IL-17 inhibitors was under four years, correlations between the type of biologics and frequency of atopic disorders are not feasible. Furthermore, we could not correctly value the role of IL-17 inhibitors on atopic conditions. However, we showed that atopic patients required more frequent switches to different biologic drugs in order to achieve the therapeutic target (1.92 TNF inhibitors compared with 1.7 in non-atopic subjects), as well as higher doses of secukinumab (300 mg monthly) in 25.5% of patients, compared with 18.8% non-atopic cases. Although statistically non-significant (*p* > 0.05), we suggest that atopic disorders may be related to difficult-to-treat SpAs. Further studies should be performed in larger cohorts of patients and with comparable exposure, in order to correctly evaluate the effectiveness of different classes of biologics on atopic conditions.

## 5. Conclusions

We successfully confirmed that atopic disorders, particularly rhinitis, atopic dermatitis, and asthma may develop in patients diagnosed with spondyloarthropathies who are taking biological therapy. Additionally, irrespective of their spondyloarthropathy, it seems that atopic patients require more frequent switching among biologics to control their active disease. Further studies are necessary to investigate if the presence of concomitant atopy could prioritize the selection of appropriate biologic drugs, in order to achieve fast and lasting disease control in patients with SpAs.

## Figures and Tables

**Table 1:** The screening 5Q questionnaire applied in patients with spondyloarthropathies.

Item/Question	YES	NO
Q1: Do you have knowledge about any atopic disorders such as asthma, eczema, or rhinitis diagnosed in/during your childhood?		
Q2: Are you currently under specific treatment for any atopic disorder such as asthma, atopic dermatitis, or rhinitis? Q3: Did you have the atopic disorder before the SpA diagnosis? Q4: Did you have the diagnosis of atopic disorder after the initiation of biological therapy for SpA? Q5: Did you have any allergic reaction to drugs, including biologics or other external chemicals?		

**Table 2:** Demographics, disease- and treatment-related parameters in study patients.

Parameter	SpA 200	axSpA 143 (71.5)	PsA *p*-Value 57 (28.5)
Gender, *n* (%) Expected total/chi-square			
Male	143 (71.5)	112 (78.3) (102.24) (0.93)	31 (54) (40.76) (2.33) 0.00 *
Female	57 (28.5)	31 (21.7) (40.76) (2.33)	26 (46) (16.24) (5.86)
Living style, *n* (%) Expected total/chi-square			
Urban	118 (59)	83 (58) (84.37) (0.02)	35 (61.4) (33.63) (0.06) 0.66 *
Rural	82 (41)	60 (42) (58.63) (0.03)	22 (38.6) (23.37) (0.08)
Age (years), *n* (%) Expected total/chi-square			
<20	4 (2)	3 (2) (2.86) (0.01)	1 (1.7) (1.14) (0.02) 0.875527 *
21–40	56 (28)	51 (35.6) (40.04) (3.00)	5 (8.7) (15.96) (7.53) 0.000132 *
41–60	91 (45.5)	69 (48.2) (65.06) (0.24)	22 (38.6) (25.94) (0.60) 0.575089 *
>60	49 (24.5)	20 (13.9) (35.03) (6.45)	29 (49.1) (13.96) (16.19) <0.00001 *
Mean age	48.7	46.2	56
Smoking status, *n* (%) Expected total/chi-square			
Smoker	45 (22.5)	40 (28) (32.17) (1.9)	5 (8.7) (12.82) (4.77) 0.00 *
Nonsmoker	155 (77.5)	103 (72) (110.82) (0.55)	52 (91.2) (44.18) (1.39)
Skeletal involvement, *n* (%) Expected total/chi-square			
Axial	158 (79)	143 (100) (120.36) (4.26)	15 (26.3) (37.64) (13.62) <0.00001 *
Peripheral	140 (70)	84 (58.7) (106.64) (4.81)	56 (98.2) (33.36) (15.37)
Inflammatory bowel disease, % (*n*)			
Expected total/chi-square	12 (6)	10 (7) (8.58) (0.24)	2 (3.5) (3.42) (0.59) 0.34 *
Duration of SpA ** Mean ± SD Median (IQR)	15.99 ± 9.49 14 (43)	15.83 ± 9.44 14 (44)	16.42 ± 9.68 14 (40) 0.90 *
Years from onset to diagnosis ** Mean ± SD Median (Range)	2.88 ± 5.49 1 (35)	2.76 ± 5.13 1 (33)	3.21 ± 6.35 0 (33) 0.97 *
Years of SpA diagnosis ** Mean ± SD Median (Range)	13.09 ± 8.94 11 (44)	12.95 ± 9 10 (44)	13.42 ± 8.87 11 (36) 0.9 *
Persistence on TNF inhibitors ** Mean ± SD Median (Range)	6.64 ± 4.03 6 (16)	6.05 ± 3.71 6 (15)	8.18 ± 4.41 8 (16) 1 *
Persistence on IL-17 inhibitors ** Mean ± SD Median (Range)	0.47 ± 0.84 0 (4)	0.44 ± 0.88 0 (4)	0.53 ± 0.53 0 (3) 1 *

* *p* calculated with chi-square calculator online: https://www.socscistatistics.com/tests/chisquare (accessed on 12 December 2021); ** parameters expressed by the mean, standard deviation (SD) and median with range calculated with OPENSTAT programs, Shapiro–Wilk, Lilliefors, and Mann–Whitney tests.

**Table 3:** Characteristics of atopic and non-atopic patients.

Parameter	Atopic Group	Non-Atopic Group	*p*-Value
	51 (25.5%)	149 (74.5%)	Significant *p* * < 0.05
Gender, *n* (%) Expected total/chi-square			
Male	36 (70.6)	112 (75.2)	
	(37.74) (0.08)	(110.26) (0.03)	0.51 *
Female	15 (29.4)	37 (24.8)	
	(13.26) (0.23)	(38.74) (0.08)	
Living style, *n* (%)			
Expected total/chi-square			
Urban	28 (55)	83 (56)	
	(28.3) (0)	(82.7) (0)	0.92 *
Rural	23 (45)	66 (44)	
	(22.7) (0)	(66.31) (0)	
Age (years), *n* (%)			
Expected total/chi-square			
<20	0 (0)	4 (2.6)	
	(1.29) (0.07)	(3.71) (0.02)	0.775064
21–40	13 (25.4)	43 (28.8)	
	(14.49) (0.15)	(41.51) (0.05)	0.643731
41–60	29 (56.8)	62 (41.6)	
	(23.54) (1.27)	(67.46) (0.44)	0.059035
>60	9 (17.6)	40 (26.8)	
	(12.68) (1.07)	(36.32) (0.37)	0.18739
Mean age	48.8	48.4	1
Smoking status, *n* (%)			
Expected total/chi-square			
	8 (15.6)	37 (23.8)	
Smoker	(11.48) (1.05)	(33.52) (0.36)	
		0.17	
Nonsmoker	43 (84.4)	112 (76.2)	
	(39.52) (0.31)	(115.48) (0.1)	
Skeletal involvement, *n* (%)			
Expected total/chi-square			
Axial	35 (73)	143 (96)	
	(39.62) (0.54)	(138.38) (0.15)	0.17
Peripheral	30 (62.5)	84 (56)	0.09
	(25.38) (0.84)	(88.62) (0.24)	
Intestinal inflammatory symptoms, (*n*) %	7 (14.5)	5 (3.3)	
Expected total/chi-square	(3.06) (5.07)	(8.94) (1.74)	0.007
Duration of disease since onset **	16.11 ± 10.06	15.94 ± 9.37	
Mean ± SD	13 (37)	14 (43)	0.99 *
Median (Range)			
Years from onset to diagnosis **	3.17 ± 5.92	2.74 ± 5.34	
Mean ± SD	1 (33)	1 (33)	0.80 *
Median (Range)			
Years of diagnosis SPA **	12.98 ± 9.40	13.16 ± 8.85	
Mean ± SD	10 (36)	11 (44)	0.87 *
Median (Range)			
Years of TNF inhibitors **			
Mean ± SD	6.93 ± 4.09	6.53 ± 4.00	0.86 *
Median (Range)	8 (15)	6 (16)	
Years of IL-17 inhibitors **			
Mean ± SD	0.48 ± 0.81	0.46 ± 0.86	
Median (Range)	0 (3)	0 (4)	0.98 *
Number of TNF inhibitors **			
Mean ± SD	1.92 ± 1.00	1.7 ± 1.00	
Median (Min, Max)	2 (1, 4)	2 (1, 5)	0.98 *
Patients on 300 mg IL-17inhibitors (*n*) %			
expected total/chi-square	13 (25.5)	28 (18.8)	
	(10.46) (0.62)	(30.54) (0.21)	0.30 *

* *p* calculated with chi-squared calculator online: https://www.socscistatistics.com/tests/chisquare (accessed on 12 December 2021); ** parameters expressed by the mean, standard deviation (SD) and median with range calculated with OPENSTAT programs, Shapiro–Wilk, Lilliefors, and Mann–Whitney tests.

**Table 4:** Distribution of patients according to the number of TNF inhibitors switched.

Number of Patients Treated with TNF Inhibitors and Number of TNF Needed						
	1 TNFi	2 TNFi	3 TNFi	4 TNFi	5 TNFi	Number of Patients
Atopy cohort	13 (22.04) (3.71)	20 (14.61) (1.98)	8 (5.51) (1.12)	4 (3.35) (0.12)	1 (0.48) (0.57)	46
Non atopy cohort	79 (69.96) (1.17)	41 (46.39) (0.63)	15 (17.49) (0.35)	10 (10.65) (0.04)	1 (1.52) (0.18)	146

The chi-squared statistic is 9.8745. The *p*-value is 0.042596. The result is significant at *p* < 0.05. Calculated with https://www.socscistatistics.com/tests/chisquare (accessed on 12 December 2021).

## Data Availability

Not applicable.
